# Enhanced Methods for Local Ancestry Assignment in Sequenced Admixed Individuals

**DOI:** 10.1371/journal.pcbi.1003555

**Published:** 2014-04-17

**Authors:** Robert Brown, Bogdan Pasaniuc

**Affiliations:** 1 Bioinformatics Interdepartmental Program, University of California Los Angeles, Los Angeles, California, United States of America; 2 Department of Pathology and Laboratory Medicine, Geffen School of Medicine, University of California Los Angeles, Los Angeles, California, United States of America; 3 Jonsson Comprehensive Cancer Center, University of California Los Angeles, Los Angeles, California, United States of America; Cornell University, United States of America

## Abstract

Inferring the ancestry at each locus in the genome of recently admixed individuals (e.g., Latino Americans) plays a major role in medical and population genetic inferences, ranging from finding disease-risk loci, to inferring recombination rates, to mapping missing contigs in the human genome. Although many methods for local ancestry inference have been proposed, most are designed for use with genotyping arrays and fail to make use of the full spectrum of data available from sequencing. In addition, current haplotype-based approaches are very computationally demanding, requiring large computational time for moderately large sample sizes. Here we present new methods for local ancestry inference that leverage continent-specific variants (CSVs) to attain increased performance over existing approaches in sequenced admixed genomes. A key feature of our approach is that it incorporates the admixed genomes themselves jointly with public datasets, such as 1000 Genomes, to improve the accuracy of CSV calling. We use simulations to show that our approach attains accuracy similar to widely used computationally intensive haplotype-based approaches with large decreases in runtime. Most importantly, we show that our method recovers comparable local ancestries, as the 1000 Genomes consensus local ancestry calls in the real admixed individuals from the 1000 Genomes Project. We extend our approach to account for low-coverage sequencing and show that accurate local ancestry inference can be attained at low sequencing coverage. Finally, we generalize CSVs to sub-continental population-specific variants (sCSVs) and show that in some cases it is possible to determine the sub-continental ancestry for short chromosomal segments on the basis of sCSVs.

## Author Summary

Advances in sequencing technologies are dramatically changing the volume and type of data collected in genetic studies. Although most genetic studies so far have focused on individuals of European ancestry, recent studies are increasingly being performed in individuals of admixed ancestry (i.e., with recent ancestors from multiple continents, e.g., Latino Americans). A key component in such studies is the accurate inference of continental ancestry at each segment in the genome of these individuals. In this work we present accurate and robust methods that use continent-specific variants (i.e., genetic variants observed only in individuals of a given continent), now readily accessible through sequencing technology, to perform extremely fast and accurate inference of the ancestral origin of each genomic segment in recently admixed individuals.

This is a *PLOS Computational Biology* Methods article.

## Introduction

Advances in high-throughput genotyping technologies have enabled large-scale studies of genetic variation, from genome-wide association studies (GWAS) [1] to inference of population history from genetic data [2]. The most notable use of high-throughput genotyping has been in GWAS where researchers have reproducibly identified thousands of genetic variants associated with many diseases [3]. Although initial studies have focused on homogenous populations [4], the development of accurate methods for discerning population structure has enabled studies across individuals of different ethnicities such as admixed populations (i.e. populations with genetic ancestry from more than one continent) [5]–[8]. Owing to their recent demographic history, admixed individuals have genomes that are a mosaic of segments originating from different continents. A key component of genetic studies in recently admixed populations is the inference of ancestry at each locus in the genome (i.e. the continental origin of each variant, local ancestry). Although local ancestry has been traditionally used to map genes to diseases through admixture mapping [8]–[10], the past few years have seen the use of local ancestry analyses in a wide range of genetic applications. Recent work has shown that admixture mapping can be used to localize missing sequences from the human reference genome [11], while other analyses of local ancestry in large samples of African American individuals have yielded novel insights into the dynamics of recombination rates across the genome [12], [13]. Local ancestry also can be leveraged to make demographic inferences from genetic data of admixed populations [14]–[17] as well as in finding signals of natural selection in African Americans [18]. Finally, local ancestry is also important for disease genetics in correcting for spurious associations in fine-mapping studies [19] as well as in finding new disease risk loci through a combination of association and admixture mapping [6], [20]–[23].

Many methods have been developed to infer local ancestry in admixed individuals. Early methods [24]–[26] relied on ancestry informative markers within hidden Markov models to achieve high accuracy. With decreasing genotyping costs, newer methods [27]–[30] were designed to use the increasing amount of data from genome-wide genotyping arrays while accounting for linkage disequilibrium (LD) among variants. The currently established methods [31]–[33] model LD in the form of haplotypes to achieve superior accuracy over non-haplotype aware approaches. Recent work in parallel to ours [34] explored the use of conditional random forests in performing local ancestry analysis. Although extremely accurate for African Americans, these methods have not achieved the same level of high accuracy in Latino Americans, partially due to the lack of good proxies for the Native American component [35] and more recent divergence among ancestral populations. Rapid cost decreases in sequencing technologies coupled with the increased power for assessing genetic variation has made sequencing the approach of choice for many of the coming genetic studies [36]–[48]. The amount of variants identified by sequencing makes local ancestry inference in large cohorts of sequenced individuals prohibitively time consuming (e.g. existing HMM-based approaches will take 5 CPU years to infer local ancestry in 15,000 sequenced African Americans, or 18 days per core on a 100-core cluster). This is particularly important as sample sizes continue to increase to hundreds of thousands of individuals [49]. For example, a recent study of obesity included over 15,000 African Americans [50] and another study included 30,000 African Americans for recombination mapping [12].

Here we present improved methods for local ancestry inference for fully sequenced admixed genomes. Sequencing, as opposed to genotyping, is able to catalogue much larger sets of variants with a large component of such variants being continent-specific (i.e. variants that are observed only in individuals from one continental group such as Europeans or Africans). For example, the 1000 Genomes Project [51] has found that 17% of variants with frequencies between 0.5–5% and 53% of variants with frequencies <0.5% are continent-specific when comparing European, African, East Asian and American populations. We hypothesized that these variants can be used for ultra-fast assignment of ancestry at every locus in the genome. We term these variants as continent-specific variants (CSVs) and model them within standard hidden Markov models of local ancestry to achieve an accurate and computationally efficient method for local ancestry inference (Lanc-CSV). Our model accounts for potential errors induced by low-coverage sequencing as well as by the finite sample size of the reference panels used for local ancestry inference. As opposed to most previous local ancestry methods that require phased reference panels, our approach only requires allele frequency information for each continental group.

Our approach is significantly faster than existing standard haplotype-based approaches making it the approach of choice for large-scale sequencing studies (e.g. our approach is able to infer local ancestry in under 42 CPU days in 15,000 sequenced genomes, or 0.42 days per core if a 100-core cluster is available). The very-fast computational speed of our approach allows it to be sample aware by iteratively improving the quality of the CSV calls using the admixed individuals themselves to further boost accuracy by eliminating spuriously identified CSVs (see Methods). We use simulations of recently admixed individuals starting from 1000 Genomes data to show that Lanc-CSV achieves comparable accuracy to existing methods (e.g. mean r^2^ = 0.92 across simulations of African Americans, Mexicans, and Puerto Ricans as compared to 0.93 for LAMP-LD [31], 0.84 for RFMix [52] and 0.80 for MULTIMIX [53]).

We investigate the effect of low coverage sequencing on our method in simulations and show that at 5× coverage our approach achieves an r^2^ = 0.86 in African Americans, 0.70 in Mexicans and 0.78 in Puerto Ricans. More importantly, we investigate whether similar results can be obtained in real data. We infer local ancestry using our approach in the real African American, Mexican, and Puerto Rican individuals from 1000 Genomes and find that Lanc-CSV agrees with the published consensus local ancestry calls [51] (mean r^2^ = 0.79 across the three sets of comparisons as compared to a mean r^2^ = 0.81 for a haplotype-based method, see Results). While our current method achieves comparable results to existing methods with the given data sets, we demonstrate that the iterative sample aware CSV updating continues to increase the overall accuracy as the sample size increases. With large studies this may give Lanc-CSV a further accuracy advantage over existing methods. Finally, we extend the concept of CSVs to sub-continental population-specific variants (sCSVs) and show that they can be used to perform ancestry assignment with individuals admixed from two ancestries from the same continent.

As the costs of sequencing rapidly decreases and genetic studies sequence more samples, the tradeoff between computational runtime and accuracy becomes critical for local ancestry inference. Using our proposed approaches we can reliably infer local ancestry in very large sequenced cohorts at a fraction of the computational cost of existing approaches. We provide Lanc-CSV as a free software package for the community interested in local ancestry inference at http://bogdan.bioinformatics.ucla.edu/software/lanccsv.

## Results

### Continent-specific variants in the 1000 Genomes data

Using data from the 1000 Genomes Project, we investigate whether CSVs can be used to perform accurate local ancestry inference. We define CSVs as single nucleotide variants in which one of the alleles is only observed in one of the continental groups (e.g. European) and absent from other continental groups. Determining CSVs can be quickly achieved using reference panels such as data generated by the 1000 Genomes Project [51]. Although extremely useful, 1000 Genomes was sequenced at low coverage (4×) with potentially many rare variants (likely to be CSVs [54]) being left uncalled. In addition, some variants are spuriously called as CSVs due to the finite sample of the reference panels; for example, variants that would be observed in larger samples from more than one continental group are mislabeled as CSVs due to the small size of the reference panels. We call these variants spurious CSVs.

We assess the presence of informative and spurious CSVs for the purposes of local ancestry inference in the real 1000 Genomes data. To mimic local ancestry inference, we used data from the TSI(97), JPT(88) and LWK(97) populations (as proxies for the European, Native American and African continental groups, numbers represent the number of individuals from each population) to infer CSVs and used a different set of haplotypes (CEU(85), CHB+CHS(197) and YRI(88)) to determine the number of observed CSVs from each continental group on a haplotype of a given group (see Methods). We observe that only a fraction of called CSVs using the reference panel are spurious in the target panel; e.g. an average of 15.70 per mega-base per chromosome of European CSVs in the reference are also observed on a target European haplotype as compared to 1.20 per mega-base per chromosome that are spuriously called (i.e. was a Native American or African CSV seen on the European haplotype) (see Table 1). The spacing between observed CSVs on a haplotype ranges on the average from ∼10 kb for African chromosomes to ∼100 kb for Asian chromosomes. Since we used data from different populations within the same continental groups (e.g. TSI and CEU), some of the European CSVs are missed as they are specific to only one population within the same continent. Therefore the numbers in Table 1 represent a lower bound on the total amount of CSVs informative for continental local ancestry inference. As previously reported, we observe a much larger number of African CSVs owing to the larger genetic diversity observed within Africa [51]. We also observe that the percentage of spurious African CSVs is much lower than that of European and Asian CSVs that are falsely identified (0.7% vs. 7.2% and 8.8%).

**Table 1:** The average number of observed CSVs per haplotype per megabase from each ancestry.

	European CSVs (TSI)	African CSVs (LWK)	Asian CSVs (JPT)
European Haplotypes (CEU)	15.70 (93%, 2.25)	1.00 (6%, 0.60)	0.21 (1%, 0.12)
African Haplotypes (YRI)	0.57 (<1%, 0.33)	123.48 (99%, 5.22)	0.33 (<1%, 0.14)
Asian Haplotypes (CHS+CHB)	0.40 (3%, 0.26)	0.64 (5%, 0.32)	10.75 (91%, 1.45)

Parentheses are the percentages of CSVs on each haplotype and the standard deviations. To estimate CSVs we used TSI, LWK, and JPT individuals as proxies for the European, African and Native American ancestries. We calculated the number of European, African and Asian CSVs seen on CEU, YRI, and CHS+CHB haplotypes. The values in parentheses represent the percentages of each ancestry type of CSV seen on a haplotype from a specific population.

### Accurate local ancestry inference using CSVs

**Figure 1:**
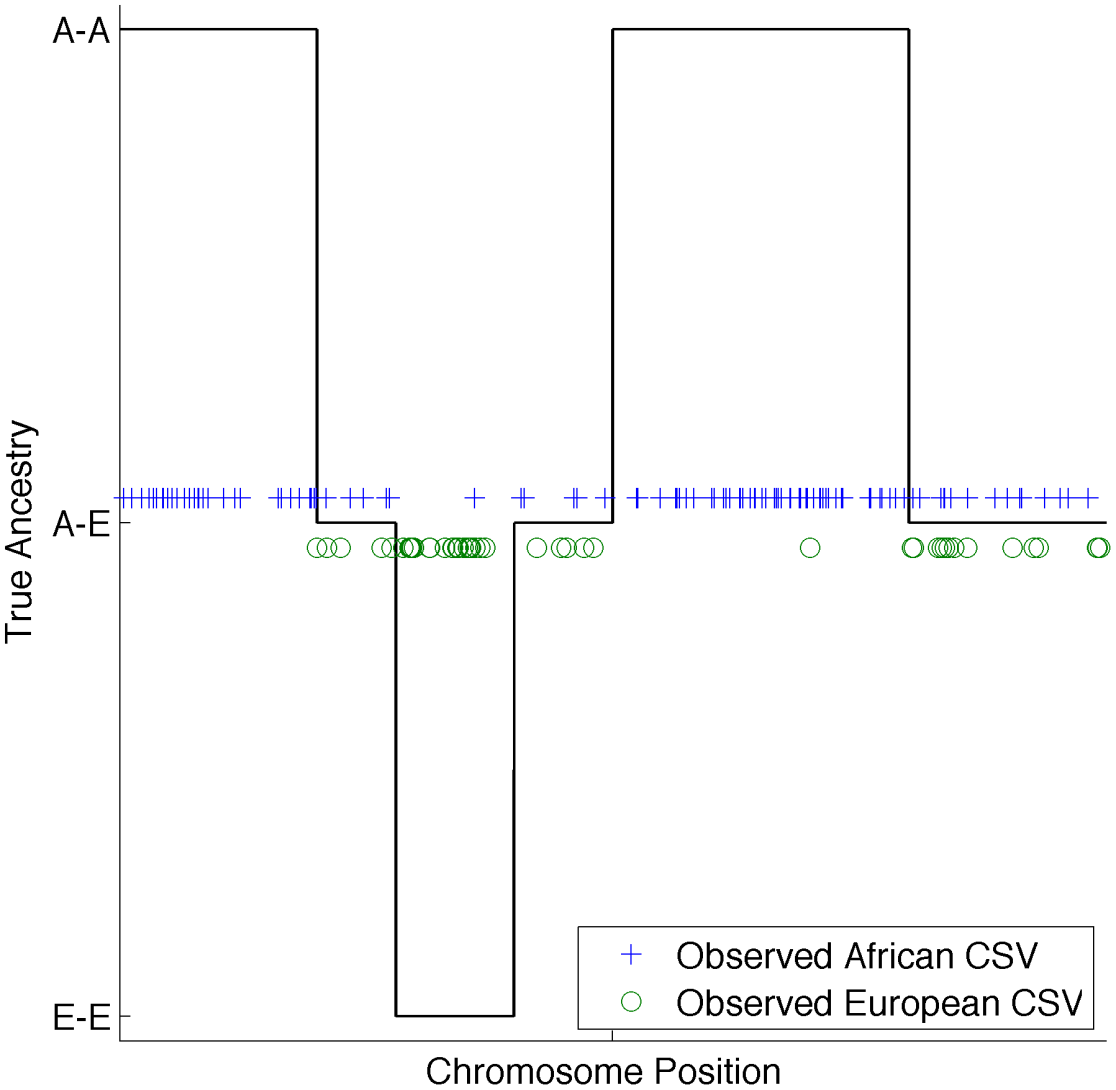
Example of CSVs in a 2-way admixed individual (e.g. African American). Lines denote the true local ancestry while the dots denote CSVs. Different dot types denote the continental ancestry of each CSV. From visual inspection it is relatively easy to discern the true ancestry from the three observed patterns. Spurious CSVs are denoted by CSVs mislabeling the true ancestry state.

The admixture process creates chromosomal segments of different ancestry in recently admixed individuals [8]. Therefore, if we visualize CSVs along the genome of a recently admixed individual, we expect to observe continuous segments with only CSVs from one continent (at loci where both alleles have the same ancestry) or a mixture of CSVs from two continents (at loci where one allele comes from one ancestry and another allele from a different ancestry) (see Figure 1). In practice we do not know the true local ancestry and we observe CSVs along the genome in an admixed individual (with potential errors) and we seek to infer the underlying local ancestry status. We extend standard hidden Markov models (HMM) for local ancestry to model CSVs as emissions and local ancestry as the underlying hidden state (see Methods). We use this model to calculate the probability of the ancestral state at each locus in the genome conditional on the observed sequence of CSVs.

**Figure 2:**
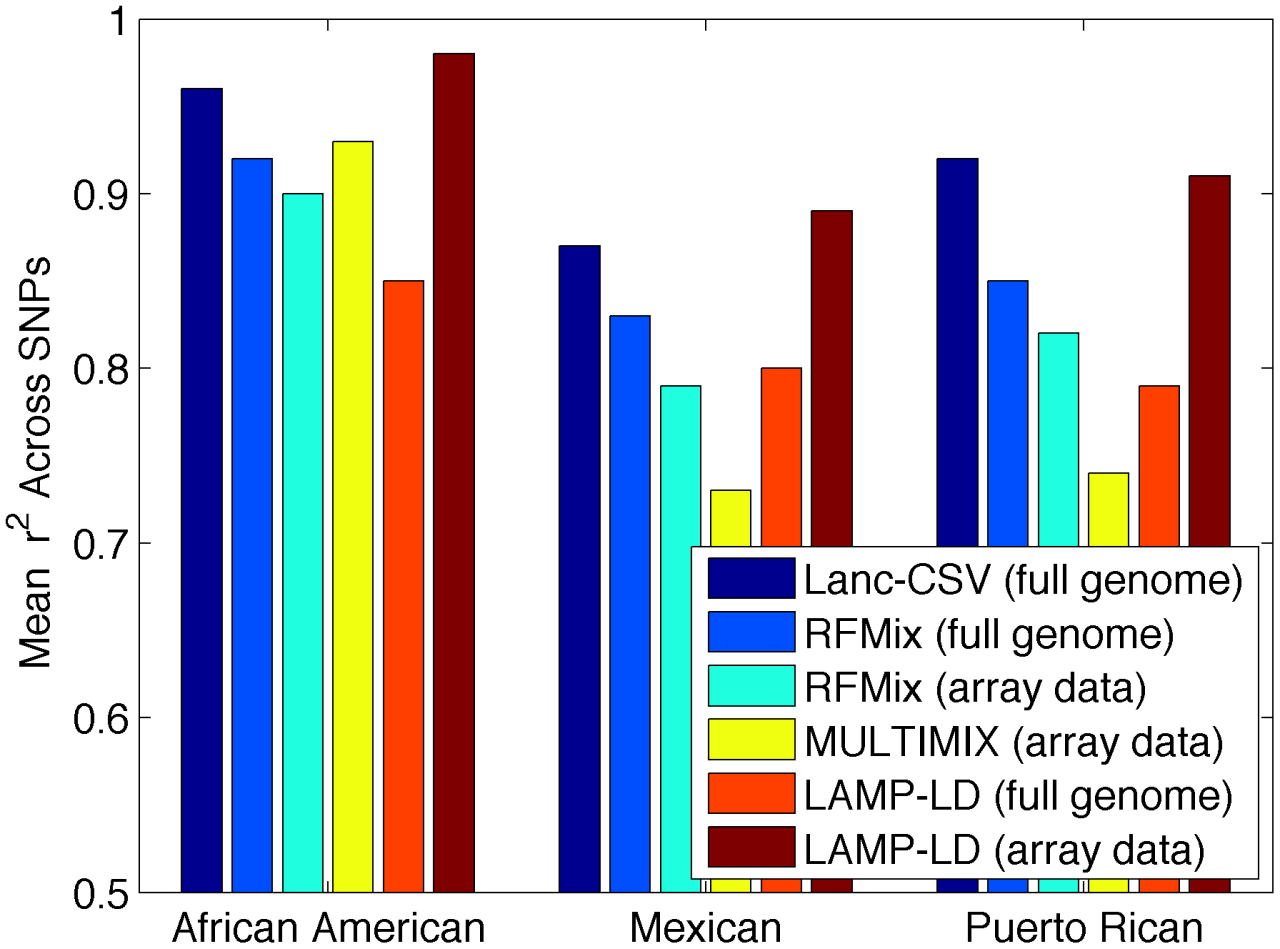
Local ancestry inference accuracy in three simulated populations. “Array data” denotes that a method was run only on the variants present on the Illumina 1 M genotyping array. “Full genome” denotes methods were run using all the variants. RFMix requires phased haplotype input, which was infered using Beagle; all other methods received unphased genotype data as input. Correlation values are the mean squared correlation across SNPs of the true vs. inferred ancestry across individuals. LAMP-LD and MULTIMIX were optimized to run with genotyping array data, possibly explaining the steep drop in accuracy when they are run using full sequencing data. MULTIMIX is not plotted when run on full sequencing data because it performed very poorly, possibly due to inaccurate parameters for sequencing data. Haploid and diploid errors are reported in Table 2.

**Figure 3:**
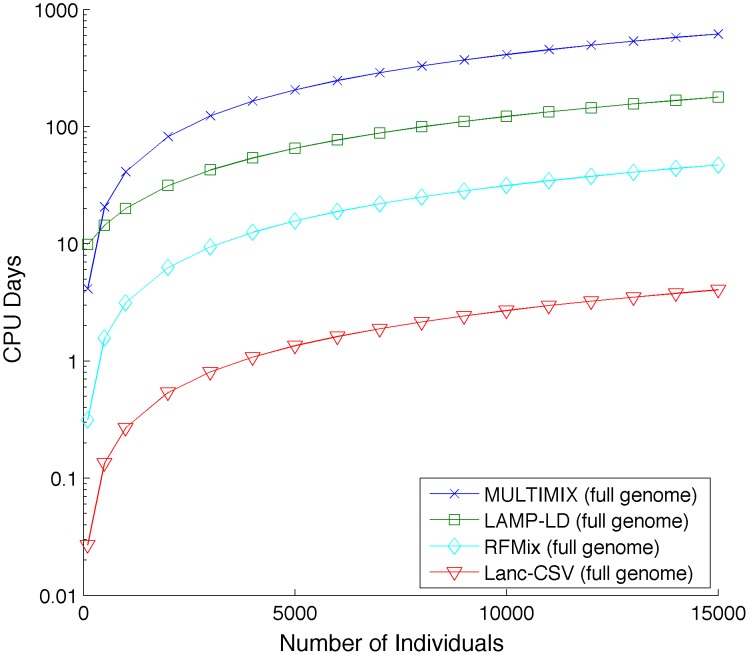
Runtime (in CPU days) as a function of the number of individuals in a study with sequencing data. Lanc-CSV is always faster than LAMP-LD and MULTIMIX when run on either full genome sequencing data or genotyping array data (see Figure S3 and Table S1). The full sequencing data contained ∼30 times more alleles than the genotyping array data. Only RFMix has comparable speed for full sequenced data and is faster for genotype array data. We show the runtime for RFMix with phasing time included.

We used simulations of African Americans, Mexicans, and Puerto Ricans to quantify the performance of our approach (see Methods). As a baseline for comparison, we used LAMP-LD and MULTIMIX, two of the fastest and most accurate methods [31], [53] for local ancestry inference. LAMP-LD models haplotypes within HMMs of haplotype diversity for ancestry assignment and has been recently shown to attain similar accuracy as another HMM-based approach (HAPMIX [32]) for African Americans and superior accuracy in Latino Americans. MULTIMIX models correlations among SNPs using a multivariate Gaussian approach; all methods utilize a window-based framework to integrate results across the genome. As a metric of accuracy, we use the squared correlation coefficient (r^2^) between the true simulated ancestry and the inferred one; the correlation coefficient measures the loss in association power for admixture mapping from errors in the local ancestry estimates [31]. We also report the percent of correctly inferred ancestry calls. Lanc-CSV attains similar results as best performing methods for ancestry inference across simulations of African Americans, Mexicans, and Puerto Ricans (e.g. mean r^2^ of 0.92 with Lanc-CSV across the considered populations)(see Figure 2 and Table 2). Interestingly, we observe that the accuracy of both LAMP-LD and MULTIMIX deteriorates when sequencing data is used; e.g. mean r^2^ of 0.93 when only SNPs on the Illumina-1M array are used, as compared to 0.71 when all sequencing data are used with LAMP-LD. Similar results are seen with MULTIMIX (see Figure 2 and Table 2). This is likely due to the fact that both LAMP-LD and MULTIMIX have been optimized for GWAS genotyping array data and not for the significant number of rare variants identified through sequencing. Recent work in parallel to ours has proposed the use of conditional random forests in local ancestry inference (RFMix [34]). We assessed RFMix accuracy on our simulations and we observe comparable accuracy as other methods for array data (see Figure 2 and Table 2). In addition, RFMix accuracy slightly increases when sequencing data is available from an r^2^ = 0.84 to 0.87. We also observe a lower performance of MULTIMIX as compared to LAMP-LD and RFMix in our simulations. The average distance between a true switch point and the inferred switch point for Lanc-CSV is 76 kb and for LAMP-LD is 91 kb, both have a standard deviation greater than 100 kb (see Figure S1). Importantly, our approach requires significantly less computational runtime than both LAMP-LD and MULTIMIX run on genotyping array data (Lanc-CSV is 3–5× faster) or sequencing data (Lanc-CSV is 40–150× faster). Lanc-CSV is slightly faster than RFMix when run on sequencing data (1.4× reduced runtime) (see Table S1 and Figures 3 and S2). However, RFMix requires phased haplotype data, which can take significant time to calculate with unrelated individuals. If phasing time is included Lanc-CSV is 12.5× faster than RFMix on sequencing data (see Table S1).

**Table 2:** Local ancestry accuracy in simulations of African Americans, Mexicans and Puerto Ricans.

	African American	Mexican	Puerto Rican
LAMP-LD (array data)	0.98 (1.00, 0.99)	0.89 (0.97, 0.93)	0.91 (0.98, 0.96)
MULTIMIX (array data)	0.93 (0.99, 0.98)	0.73 (0.94, 0.80)	0.74 (0.93, 0.86)
RFMix (array data)	0.90 (0.98, 0.97)	0.79 (0.93, 0.87)	0.82 (0.95, 0.91)
LAMP-LD (full genome)	0.85 (0.97, 0.95)	0.80 (0.94, 0.89)	0.79 (0.95, 0.90)
MULTIMIX (full genome)	0.46 (0.84, 0.72)	0.44 (0.73, 0.49)	0.40 (0.74, 0.56)
RFMix (full genome)	0.92 (0.99, 0.97)	0.83 (0.95, 0.89)	0.85 (0.96, 0.92)
Lanc-CSV	0.96 (0.99, 0.99)	0.87 (0.96, 0.92)	0.92 (0.98, 0.96)

Accuracy is reported as mean r^2^ (haploid accuracy, diploid accuracy). “Array data” denotes that a method was run only on the variants present on the Illumina 1 M genotyping array. “Full genome” denotes methods were run using all the variants. RFMix requires phased haplotype input that was phased using Beagle; all other methods received unphased genotype data as input. Correlation values are the mean squared correlation across SNPs of the true vs. inferred ancestry across individuals. Accuracy is reported as mean r^2^ (haploid accuracy, diploid accuracy). LAMP-LD and MULTIMIX were optimized to run with genotyping array data, possibly explaining the steep drop in accuracy when they are run using full sequencing data.

### Extension to low-coverage sequencing

**Figure 4:**
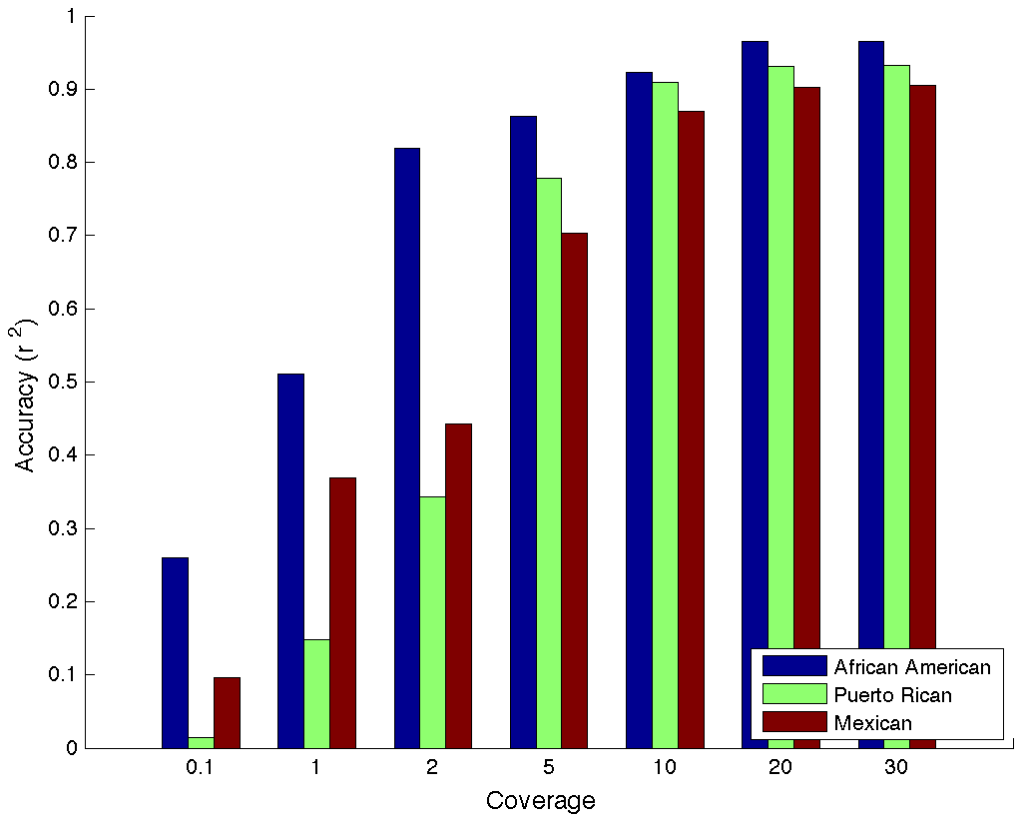
Accuracy as a function of sequencing coverage. African-Americans with only two distinct ancestral populations increases fastest in accuracy.

Recent works have shown that low-coverage sequencing yields superior association power per unit of cost as compared to genotyping arrays in GWAS [55]. The accuracy of genotype calling from sequencing data is directly related to the read coverage. High read coverage increases the likelihood of observing true CSVs, while low read coverage increases the likelihood of both not observing a CSV and spurious CSVs due to errors in the genotype calling from read data. We extend our method to low-coverage sequencing data by means of a preprocessing step where a CSV is called present at a locus if the genotype dosage (i.e. the expected count of alternate alleles given the observed reads) is above a set threshold level at a CSV location (see Methods). We estimate the genotype dosage from reads using standard techniques (see Methods). Through simulations, we determine that the Wahlund Effect [56], [57] is likely not going to impact our assumptions of Hardy-Weinberg Equilibrium at the allele frequencies of most CSVs (see Methods and Table S3). Starting from the previous simulations, we simulated sequencing data at various coverages using standard parameters for sequencing (see Methods). At 5× coverage we observe an accuracy of 0.86 for African-Americans, 0.70 in Mexicans and 0.78 in Puerto Rican simulations. As expected accuracy increases as coverage increases with little gains in accuracy coming above 10× (e.g. an accuracy of 0.91 at 10× in Puerto Rican simulations) (see Figure 4).

### Sample-aware inference of local ancestry improves accuracy

Accurate methods for local ancestry inference leverage reference panels of haplotypes to use as proxies for the missing ancestral individuals that mixed to form current admixed populations. Recent works have shown that local ancestry inference can be improved when using the admixed samples themselves to rebuild the reference panels of haplotypes [35]. A major advantage of our approach for local ancestry inference is that we can iteratively re-estimate CSVs by incorporating information from the inferred ancestry regions in the admixed samples themselves. In particular, we first estimate CSVs using external reference panels of haplotypes (e.g. 1000 Genomes), then call local ancestry and in an iterative fashion, re-call CSVs using confidently called ancestry segments from the sample itself (see Methods). This procedure reduces the number of spuriously called CSVs while determining new CSVs and increasing the overall accuracy of the method. In addition, this allows for sample-aware reference panels that are better proxies for the true ancestral population of current day admixed individuals. For example we observe an increase in accuracy from r^2^ = 0.87 to 0.92 in 200 simulated admixed Puerto Ricans after four iterations. The greatest increase in accuracy is after the first iteration and very little increase in accuracy comes with the fourth iteration. The main source of errors in Mexicans and Puerto Ricans is in distinguishing European and Native American regions that have a much lower signal to noise ratio than in African and European or African and Native American regions (see Table 1). African American inference is highly accurate at all sample sizes because even without any iterations, accuracy is high due to the strong signal to noise ratio allowing African and European segments to be easily distinguished.

**Figure 5:**
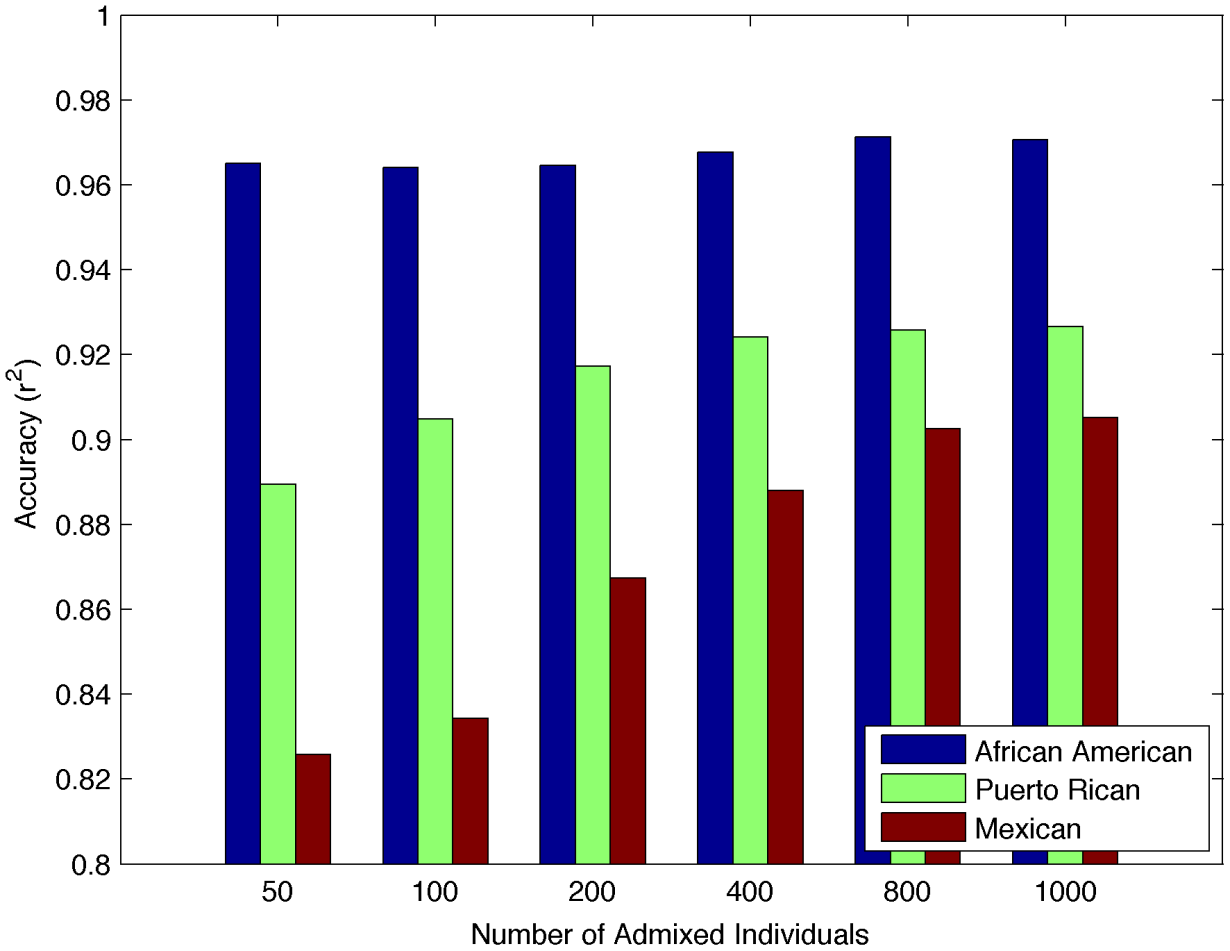
Accuracy as a function of sample size. While accuracy increases with increasing numbers of admixed individuals, the most significant increase is seen in Mexican individuals. We report accuracy for Lanc-CSV using 200 admixed individuals, but accuracy exceeds this as the number of admixed individuals increases. This is due to the method being better able to correct for spurious CSVs and to add in new CSVs when there are more individuals.

As compared to previous methods that do not use information from the other admixed individuals when calling local ancestry, Lanc-CSV will continue to increase in accuracy as the admixed sample size increases. Figure 5 plots the accuracy as a function of the number of admixed individuals. As expected we observe that the accuracy increases as the number of individuals increases with 200 samples being sufficient for high accuracy comparable to LAMP-LD in these simulations. However, as more simulated samples are added in, accuracy exceeds that of LAMP-LD in both the Mexican and Puerto Rican ancestries.

### Analysis of real admixed individuals from 1000 Genomes

We investigated whether similar results can be achieved in real admixed genomes. We used our approach and LAMP-LD to call ancestry in the real data from the Americans of African Ancestry in South Western USA (ASW), Mexican Ancestry from Los Angeles (MXL), and Puerto Ricans from Puerto Rico (PUR) individuals from the 1000 Genomes Project. 1000 Genomes provided local ancestry calls for these individuals based on the consensus of four current local ancestry inference methods [31], [32], [52], [53]. Since the true ancestry is not known for these individuals, we measured the correlation between the local ancestry calls of our approach with the ancestry calls provided by 1000 Genomes. We observed an average correlation rate (r^2^) on chromosome 10 of 0.94, 0.63, and 0.81 for Lanc-CSV and 0.99, 0.66 and 0.79 for LAMP-LD (which was used as part of inferring the consensus calls) in African Americas, Mexicans and Puerto Ricans respectively (haploid and diploid errors reported in Table S2).

These low r^2^ values are likely a result of poor reference panels in our inference since we are using the Asian haplotypes as proxy for Native American panels (1000 Genomes project used a specially designed panel for Native Americans [58]). To further investigate this hypothesis, we used our method to infer local ancestry in 20 of the Mexican individuals using the rest of the Mexicans as reference panel (that is, we used the consensus ancestry calls provided by 1000 Genomes to call CSVs). We observe a large increase in accuracy when incorporating the other Mexicans (and their local ancestry consensus calls) in the reference panel (mean r2 of 0.80 versus 0.66 if only Asian samples are used as reference).

This demonstrates that the low accuracy of both LAMP-LD and Lanc-CSV in real data is likely due to poor reference panels. It also demonstrates that a sample aware method could overcome this obstacle if sufficient admixed individuals are available. Therefore, we use the consensus calls of the Mexicans and Puerto Ricans of the 1000 Genome data to build improved CSV reference panels and provide them as a free resource to be used with Lanc-CSV for new sequenced admixed individuals.

### Sub-continental ancestry calling

**Figure 6:**
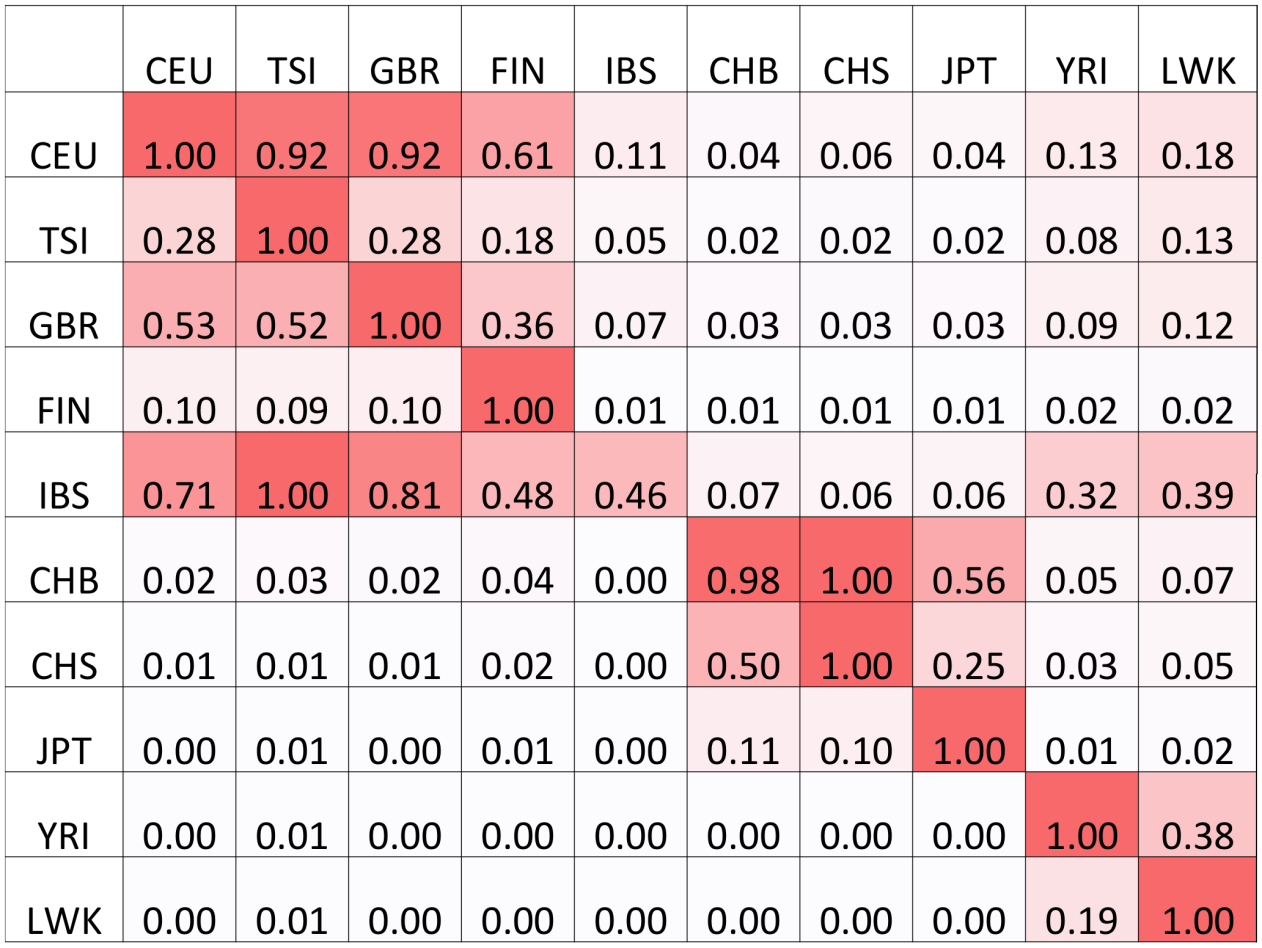
Proportions of sCSVs from each population observed on a held out haplotype. Each row represents the ancestry of the haplotype that was held out and each column represents the average number of sCSVs observed on the held out haplotype from the given population. Each row is normalized by the maximum value of the row so that the population with the most sCSVs observed has a value of 1. In each row, higher values are associated with populations in the same continental group as would be expected. The IBS have only fourteen individuals, which makes determining IBS sCSVs extremely difficult.

We extend continent-specific variants to sub-continental population-specific variants (sCSVs). We define sCSVs as variants that are observed in only one of the 1000 Genomes populations and not in any other (e.g. a variant observed only in the individuals from Great Britain (GBR) and never in any of the other populations). Using a leave one out analysis we demonstrate in Figure 6 that the chromosomes from 9 out of 10 populations have more observed sCSVs from the population from which it was observed than from any other. Due to limited reference panel size and the closeness of the sub-continental populations, there are considerable numbers of spurious sCSVs, but not enough to make sub-continental ancestry calling impossible in some scenarios. The two exceptions are the IBS population that only has 28 reference haplotypes (not enough to accurately determine sCSVs) and the CHB and CHS that are genetically very similar.

**Figure 7:**
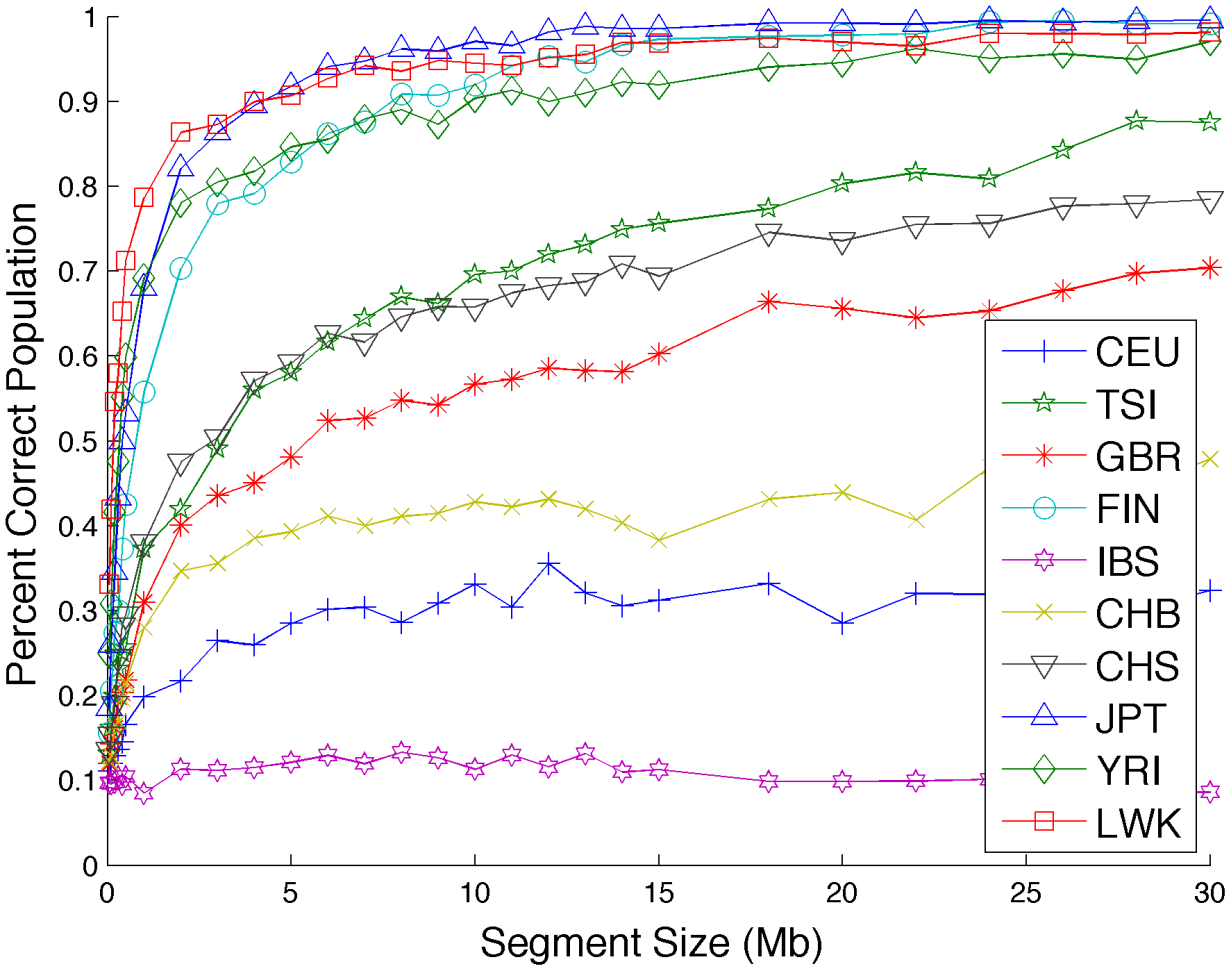
sCSVs allow for calling the sub-continental population of a haplotype. Randomly drawn segments of haplotypes from known populations can be accurately assigned to the population of origin. Accuracy for each population is significantly correlated with the number of reference haplotypes for that population (r = 0.65, p-value = 0.042). The highest accuracies are seen in populations that are more isolated from other populations in their continents.

**Figure 8:**
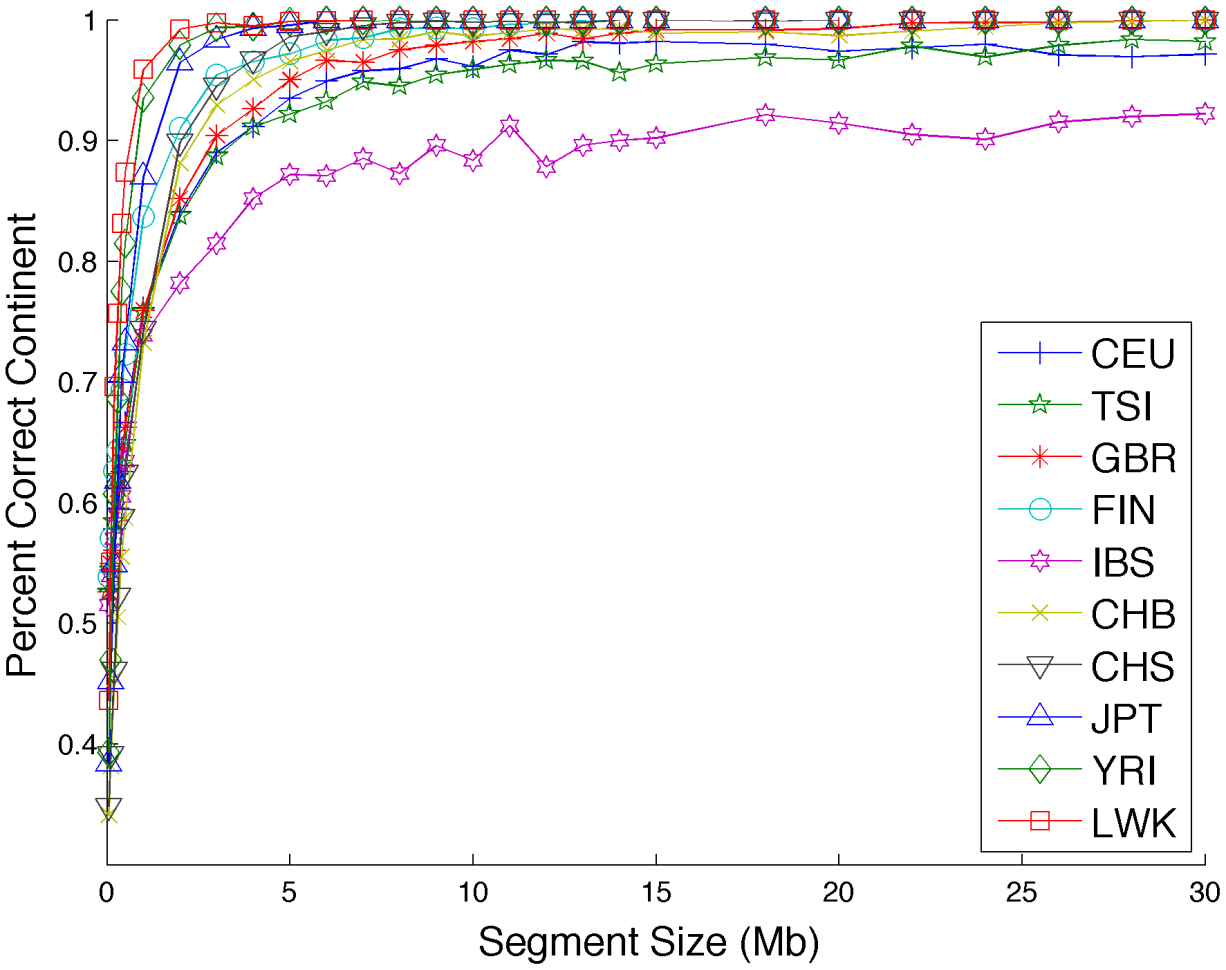
sCSVs are able to assign the correct continental group to small haplotype segments with high accuracy. This shows most of the incorrectly called accuracies still call to the correct continental group.

We assess the ability of correctly calling the population through a leave-one-out procedure starting from the real 1000 Genomes haplotype data. For each held out haplotype we randomly select short segments between 0.1 and 30 megabases and assign them to the population that has the maximum sCSV count across this segment. We plot the accuracy of this naïve calling as a function of segment size in Figure 7. We also calculate the accuracy of assigning each haplotype to the correct continental group based on sCSVs in Figure 8. Correlating the accuracy of assigning the correct population to the haplotype segment (length 10 megabases) with the size of the reference panel of the called segment achieves a correlation coefficient of r = 0.65 (p-value = 0.042) showing that larger reference panel sizes are associated with more accurate sub-continental population ancestry inference. The two African populations (YRI and LWK) as well as the Finnish (FIN) and (JPT) have very accurate ancestry calls possibly due to a higher degree of genetic differentiation as compared to the other sub-continental populations. The CEU individuals are Utah residents with northern and western European ancestry and may already be sub-continentally admixed which could potentially explain the low accuracy seen with the CEU. The IBS do not have enough reference panels to be able to call IBS sCSVs. As expected, when errors are being made, most of the errors resulted in another population from the same continental group being called (Figure 8). We also simulated diploid admixed individuals from pairs of sub-continental European populations with moderate accuracy in Lanc-CSV (Table S4).

**Figure 9:**
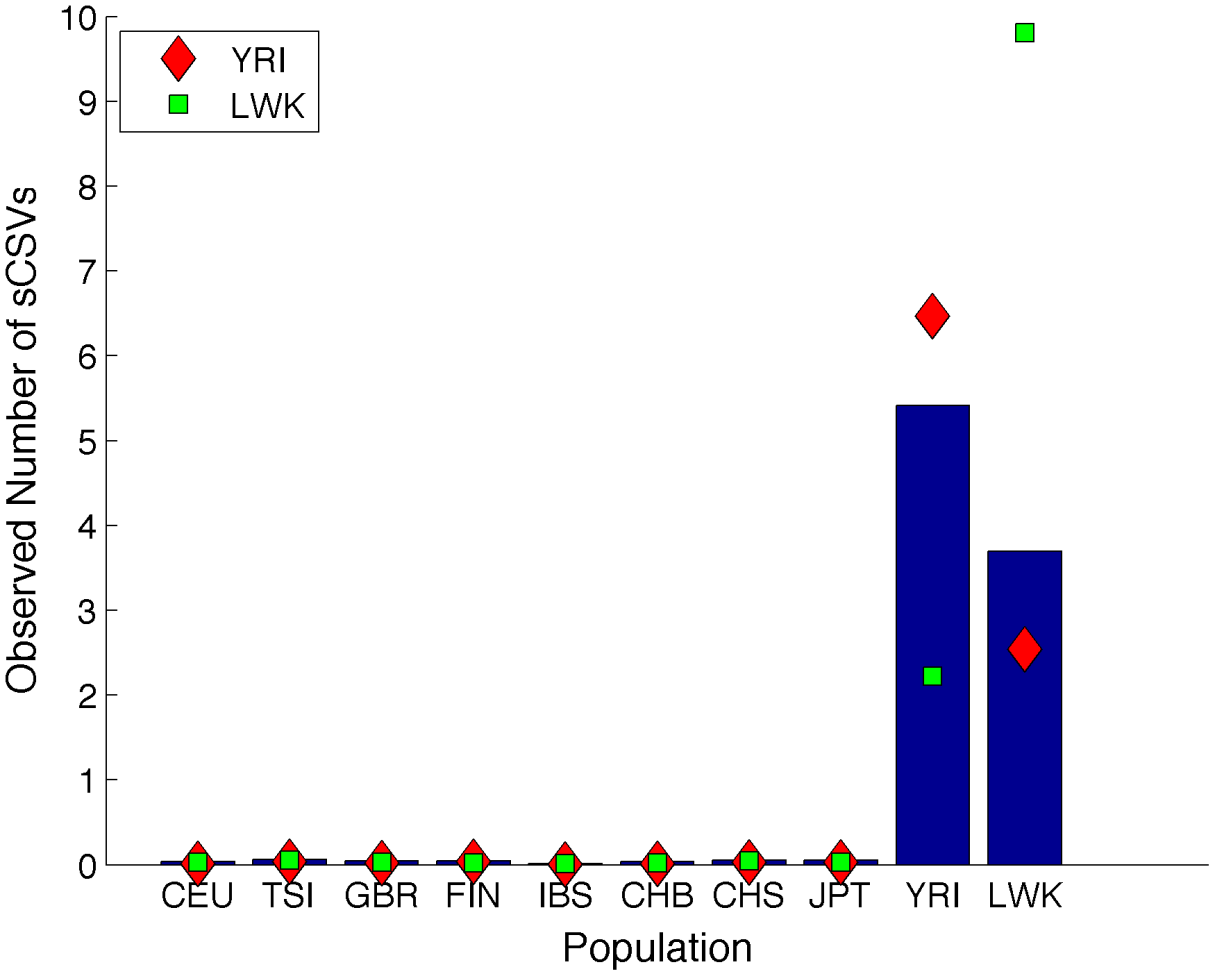
The average number of sCSVs from each 1000 Genomes population observed per megabase on the African-African called local ancestry regions of the real ASW individuals on chromosome 10. The large number of YRI sCSVs seen in these regions supports the hypothesis that the African admixture component in African Americans comes from western Africa. We plot the expected number of observed sCSVs per megabase on a YRI haplotype (red diamonds) and the expected number of observed sCSVs on an LWK haplotype (green squares). The observed counts more closely resemble the count profile expected from the YRI haplotypes.

**Figure 10:**
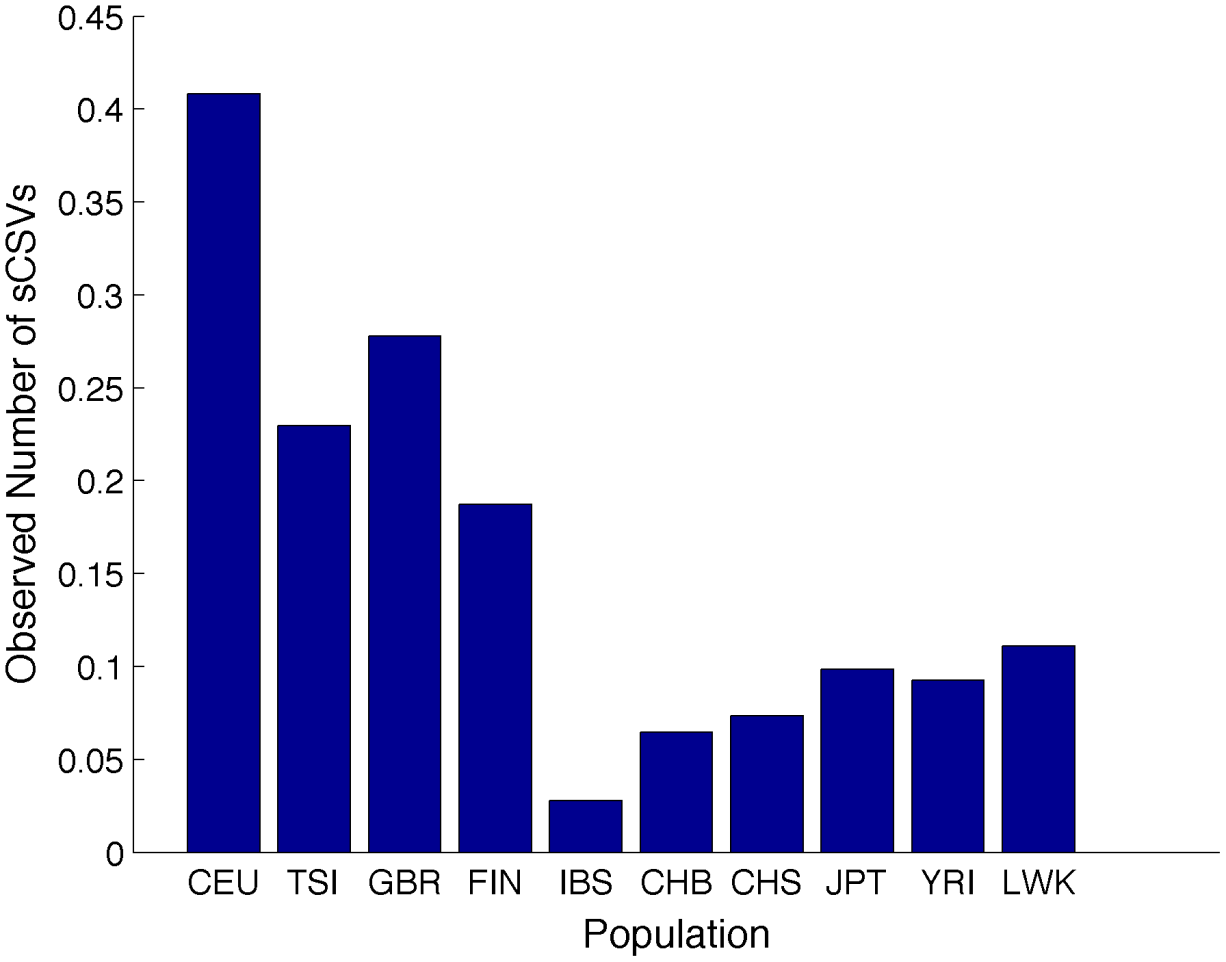
The average number of sCSVs from each 1000 Genomes population observed on the European-European called local ancestry regions of the real ASW individuals.

In order to determine the effectiveness of the sCSV approach in real data, we counted the sCSVs observed per megabase in African-African and European-European continental called ancestry regions of the ASW individuals on chromosome 10 (using the 1000 Genomes consensus local ancestry calls). Figure 9 shows that in the African-African regions there is strong enrichment for YRI sCSVs. We additionally plot the expected number of observed sCSVs on a YRI haplotype (red diamonds) and the expected number of observed sCSVs on an LWK haplotype (green squares). The observed counts more closely resemble the count profile expected from the YRI haplotypes. This supports the established hypothesis that the African component of the ASW is likely from western Africa [15]. When looking at the European-European segments of the ASW (Figure 10), the most sCSVs are CEU followed by GBR supporting the hypothesis that the European ancestors are more related to northwestern Europeans. However given the small admixture proportion of European ancestry in African Americans, there are only a few small regions of European-European ancestry resulting in the very low sCSV counts for the ASW in these regions as compared to the African-African ancestry regions.

## Discussion

We have presented here an approach for local ancestry inference in fully sequenced recently admixed individuals. Our approach makes use of alleles that are found to be present only in individuals from a given continental group (continent-specific variants, CSVs). Through the use of real data from 1000 Genomes we have shown that the density of such CSVs is high enough across the genome to allow for fast and accurate inference of local ancestry. It should be noted that the 1000 Genomes haplotypes are based on 4× sequencing data. Not only does the low coverage make this data noisier, but it also misses many CSVs that are in the individuals but not called due to the low coverage. As more high coverage reference panels are constructed our method will become increasingly accurate as more CSVs are identified and spurious CSVs removed. Having no pre-compute time and fast runtime per individual allows for our approach to be sample-aware in an iterative fashion. As opposed to previous approaches Lanc-CSV shows increased accuracy as more admixed samples are being analyzed. We show that as the method is run on increasing numbers of simulated individuals, it exceeds the accuracy that is obtained by LAMP-LD on the 200 Mexican and Puerto Rican samples. We expect this feature to become more important as larger sample sizes are being analyzed since the accuracy should continue to increase.

The real data analysis demonstrates the necessity of having reference panels well matched to the admixed population or having a sample aware method that can correct for poorly matched reference panels. Lanc-CSV achieves comparable results to LAMP-LD in these few real African-American, Puerto Rican and Mexican individuals. Unlike LAMP-LD, we expect our approach to continue improving in accuracy as more sequenced individuals from each continental population become available. We extended the concept of continent-specific variants to sub-continental population-specific variants and showed that under some scenarios it is possible to determine the sub-continental ancestry. We confirmed that in real ASW individuals, admixture was most likely between individuals from western Africa (near or related to the YRI); as more reference panels become available for these and other populations, we expect sCSVs to be increasingly informative of the sub-continental population ancestries. Although sCSVs show potential for sub-continental ancestry calling in haploid data, more sophisticated methods may prove fruitful for diploid calling.

A future direction for research that may prove fruitful is to relax assumptions used in our approach and by finding better ways to parameterize the method. Linkage disequilibrium among the CSVs is a main contributor to errors and further work explicitly modeling the LD structure between CSVs may provide increased accuracy. The current method has a uniform error rate for spurious CSVs across all ancestries. However the number of spurious European CSVs is much higher than the number of spurious African CSVs and the number of reference haplotypes is not controlled for in determining the error rate; therefore a non-uniform error model may further increase accuracy.

## Methods

### Data and simulations

The 1000 Genomes Project [51] has produced a public catalog of human genetic variation through sequencing in individuals from populations across the world. In this work we use the 88 Yoruba (YRI) and 97 Luhya (LWK) individuals as proxy for the African haplotypes; the 85 Utah residents with northern and western European ancestry (CEU) and 97 Tuscans in Italy (TSI) individuals were used as proxy for the European haplotypes; the 88 Japanese in Tokyo (JPT), 97 Han Chinese in Beijing (CHB) and 100 Southern Han Chinese (CHS) individuals were used as a proxy for the Native American haplotypes. The 14 Iberian populations in Spain (IBS), 93 Finnish in Finland (FIN) and 89 British in England and Scotland (GBR) individuals are also used for determining sCSVs. We used the 1000 Genomes phased haplotypes from each individual. We restricted our analysis to chromosome 10. The TSI, JPT and LWK haplotypes were used as training haplotypes for CSVs and all of the CEU, CHB+CHS and YRI haplotypes were used as simulation haplotypes so that the training and simulation haplotypes would be disjoint and unmatched. Following previous works we filtered A/T and C/G variants from the analysis [35] leaving 1,581,313 (50,000) SNPs used for sequencing (array) simulations.

Similar to previous works [32], we simulate admixed chromosomes as a random walk over the 1000 Genomes haplotypes. Distance to the next crossover is sampled from an exponential distribution with parameter 1/(λG) where λ = 10^−8^ base pairs per generation and G is the number of generations since admixture [31], [32]. At a crossover event, an ancestry (i.e. continental group) is chosen according to admixture-specific proportions and a random haplotype is drawn uniformly from that continental group. We simulate 2000 haplotypes this way and paired them to form 1000 genotypes with no simulated haplotype used more than once. We used the following admixture proportions (θ) for the European, Native American and African ancestry: 0.45∶0.5∶0.05 for Mexicans and 0.67∶0.13∶0.2 for Puerto Ricans and 0.2∶0.0∶0.8 for African-Americans [58]–[61]. For African Americans we simulated data assuming 6 generations since admixture (G = 6) and for Mexicans and Puerto Ricans we assumed 15 generations (G = 15).

### Continent-specific variants in the 1000 Genomes data

Comparing sequenced samples from different continental groups identifies continent-specific variants. A CSV is identified if the reference or alternate allele is observed in only one of the continental groups being compared. A CSV is only informative of an individual's ancestry if it is observed in that individual. We used the reference panels to estimate CSVs and then identified how many European, Native American, and African CSVs per megabase per haplotype are present in the haplotypes used for simulations. That is, we count the total CSVs from each group observed in the simulation haplotypes of given group and normalize by sample size and chromosome length. The expected number of informative CSVs per megabase per haplotype gives an indication of how well local ancestry can be inferred using only CSVs.

### Local ancestry inference using CSVs

Following previous works [28], we consider admixed populations arising from *K* ancestral populations A_1_,…,A*_K_* that have been mixing for G generations. For a given admixed genotype from the admixed population, we describe each individual genotype as a vector *g*, where g*_i_*∈ (0,1,2) is the number of alternative alleles of that individual at SNP *i*. At position *i*, the individual's two alleles have either both descended from the same ancestries (i.e. continental group) or from two different ancestries. We are interested in determining the ancestry origin of the two alleles at each position *i* in the genome. Our model is based on an HMM described by a triple H = (Q,δ,ε), where Q is the set of states, δ is the transition probability function and ε is the emission probability function. A different HMM is estimated for each individual at each iteration with parameters estimated from the locations of informative CSVs in each individual.

We denote by Q each possible combination (including the same ancestry) of ancestries in a diploid genome. The transition function δ changes at each step *j* as a function of the genetic distance between informative CSVs. The emission probabilities ε are constant for each state in Q. For any number of ancestral groups K, there are nine transition types that are typical of all possible transitions (not all are needed if K<4)(see Table 3). The transition functions described can describe the transition from any state *q* at step *j*-1 to any state *q′* at step *j* (see Figure S2). Here r is the probability of one or more recombinations occurring between the *j-1* ^th^ informative CSV and the *j* ^th^ informative CSV and is a function of the genetic distance between the two of them. This is modeled as a Poisson process with parameter dGλ as the probability of one or more recombinations occurring between two SNPs separated by distance d, having recombined G generations ago and with a rate parameter λ. There is significant linkage between many of the CSVs so we set λ = 10^−15^ in order to minimize the effect of close highly linked CSVs.

**Table 3:** The transition probabilities between ancestry pairs.

	(A_1_,A_1_)	(A_1_,A_2_)	(A_3_,A_3_)	(A_2_,A_3_)	(A_3_,A_4_)
(A_1_,A_1_)	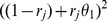	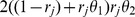	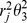	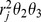	NA
(A_1_, A_2_)	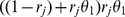	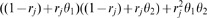	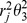	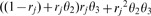	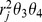

If A_k_ represent a specific ancestry and θ_k_ represents the admixture proportion of that ancestry in the admixed population, then these equations are the transition probabilities for all possible types of transitions given a probability r*_j_* of one or more recombinations occurring between the previous informative CSV and the *j* ^th^ informative CSV. The rows represent the ancestry state at the previous CSV and the columns the ancestry state being transitioned into at the *j^th^* CSV.

It is impossible to perfectly determine which CSVs are spurious from the reference sets, so emission probabilities must reflect the possibility of errors in determining which variants are continent-specific (see Table 4). In a section of heterogeneous ancestry, emissions from the two ancestries are expected to occur proportional to the expected number of informative continent-specific variants seen in the two ancestries. In a section of homogenous ancestries, emissions are expected from only the one ancestry. We assume a low spurious CSV rate of ε_CSV_ = 10^−5^ and allow for the iterations to correct for errors by removing spurious CSVs identified in confidently called homozygous ancestry sections. We assume that the first state (q_0_) of the HMM is silent. With the HMM defined for each individual, the probability of the individual's continent-specific variants is computed by summing over all paths 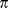 of length L (the number of CSVs showing alternate alleles in that respective individual):
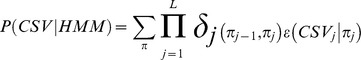



**Table 4:** Probability of emitting an informative CSV from an ancestry state.

	π = A_1_A_1_	π = A_1_A_2_
CSV = A_1_	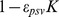	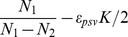
CSV≠A_1_	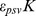	NA
CSV = A_2_	NA	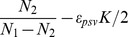
CSV = A_3_	NA	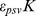

The probability of seeing a CSV from a different ancestry in a homozygous ancestry state is *ε*_CSV_. In heterozygous states, CSVs are expected to be observed proportional to the ratio of the expected number of informative CSVs per haplotype per megabase per individual (see Table 1) in the two populations. N_k_ represents the expected number of informative CSVs per haplotype per megabase per individual in population k.

The HMM is posterior decoded and local ancestry is called by assigning each CSV location the ancestry pair that had the highest posterior probability. Ancestry was called at all variants by calling a variant's ancestry as the same as the proceeding informative CSV.

Once ancestry calls have been assigned, reference panels are updated to reflect newly identified CSVs and to remove spurious CSVs. For each reference continental group k and for each allele *i* in the genome, the sample allele frequency *p_ki_* is found by summing the alternate allele count across all individuals at allele i with homozygous ancestry for group k at that allele and then dividing by twice the number of homozygous calls at that locus for group k. This is performed for all homozygous ancestry SNP locations in individual *i* except for at SNPs that are within 10 SNPs of an ancestry transition since these are likely to be less confidently called. The minor allele frequency 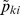, first calculated from the reference haplotypes is then updated.
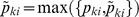



The maximum value is used because allele frequencies are used as indicators of the presence of a CSV in a population; that the frequency is equal to zero or greater than zeros is what is important for training CSVs. Another iteration of posterior decoding is performed using this new 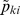 in order to determine CSV locations. We generally observed negligible improvement in accuracy between the 3^rd^ and 4^th^ iteration.

### Comparison to array-based methods

We compared Lanc-CSV to LAMP-LD (v1.0) and MULTIMIX (v1.1.0), two widely used state-of-the art methods for local ancestry inference and a concurrently published method, RFMix (v1.0.2). We used unphased genotype data as input for all methods except RFMix, which requires phased haplotypes. We ran LAMP-LD using the same parameter settings used by 1000 Genomes [51] (number of states 25 and window size 100). We ran RFMix using the default settings with no EM iterations because of the large reference panel sizes. RFMix must be used with phased haplotypes that we computed with Beagle [62] using 30 haplotypes each from the African, European and Native American (Asian) reference panels as haplotype references for phasing. We ran MULTIMIX using the MULTIMIX_MCMCgeno method (which cannot be run with the resolve step). We ran it using the suggested misfit rates for two-way admixture [0.95 0.05; 0.05 0.95] and [0.95 0.025 0.025; 0.025 0.95 0.025; 0.025 0.025 0.95] for three-way admixture. For sequencing results we passed the fully sequenced reference haplotypes and the 200 simulated admixed individuals' data to the programs. For array-based results we passed only the data at variants present on the Illumina 1 M genotyping array (down sampled randomly to 50,000 variants in order to run on LAMP-LD). We parallelized LAMP-LD, RFMix and MULTIMIX for the fully sequenced data by splitting the data into small segments (∼50,000 SNPs per segment) across the chromosome. We computed accuracies by correlating the true and inferred local ancestry at each SNP across individuals only at the Illumina 1 M chip variants.

### Low-coverage sequencing

Using the same 200 genotypes for each simulated admixed population as above, we simulate read data for each individual. We assume that the number of reads covering each variant in each individual is drawn from a Poisson distribution with the rate parameter set to the average read coverage across the genome. We simulate reads for 0.1×, 1×, 2×, 5×, 10×, 20×, and 30× average coverage across the genome.

We adapted the inference method above to function with input read count data instead of genotype data. Given a set of read data for an individual at SNP *i*, r_i_ = (ref_i_,alt_i_), where *ref* and *alt* are the counts of reads of the reference allele and the alternate allele. We first compute genotype dosage (d_i_) at SNP location *i* using the admixture proportion weighted mean frequency in admixed individuals (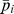) of the alternate allele. Let ancestral population *k* have admixture proportion *θ_k_* on average in the admixed individuals.
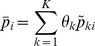



Then P(g_i_), where g_i_ is the genotype, is assumed to follow Hardy-Weinberg Equilibrium with alternate allele frequency 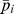. *P*(*r_i_*|*g_i_*) follows a binomial distribution modeling the number of alternate alleles seen given the number of trials equal to the total number of reads and the probability of an alternate allele equal to 1-*ε_s_*, 0.5 and *ε_s_* for g = 0, 1, or 2. We assume a sequencing error rate of *ε_s_* = 0.01. We then calculate the genotype dosage:
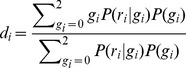



When *d_i_*>0.6, we assume that the alternate allele is present at position *i* and can then run Lanc-CSV as previously described. We choose this threshold value so that the false positive rate is below 0.0025 and the false discovery rate of observed CSVs is below 0.2 for all coverage levels at or above 1×.

The Wahlund Effect [56], [57] decreases heterozygosity and breaks Hardy-Weinberg Equilibrium when individuals from multiple populations are sampled and have different allele frequencies. CSVs are very rare and 98% of CSVs have an allele frequency <5% in the population in which they are observed. In order to ensure that the Wahlund Effect does not significantly affect our method, we calculate the probability of each genotype for a CSV with frequency 5% in one population admixing with another population with CSV frequency 0%, where the admixture proportion of the population with the observed CSV is 10%, 50% and 80% (see Table S3). The magnitude of the effect is decreases as CSV frequency decreases so these are the most extreme expected values. The effect also assumes that sampled populations have not mixed, so each generation since admixture will further decrease the effect size.

### Effect of sample aware inference

In order to determine if the accuracy of Lanc-CSV increases with increasing numbers of admixed individuals, we used an additional 800 African Americans, Puerto Ricans, and Mexicans each giving a total of 1000 simulated admixed individuals for each population. We then run Lanc-CSV for 50, 100, 200, 400, 800 and 1000 individuals in each sample and compute r^2^ after 4 iterations. Each set of individuals contains the individuals from smaller data sets. Figure 5 shows the increasing accuracy with increasing sample size.

### Analysis of real admixed individuals from 1000 Genomes

In order to assess the performance of our approach in real data, we used the Americans of African Ancestry in South Western USA (ASW), Mexican Ancestry from Los Angeles (MXL), and Puerto Ricans from Puerto Rico (PUR) genotypes from real individuals contained in 1000 Genomes. Since the true ancestry is not known, we evaluate the accuracy of Lanc-CSV by comparing to the local ancestry calls provided by 1000 Genomes. The 1000 Genomes calls are the consensus calls of four established local ancestry methods (including LAMP-LD). Calls were made at a locus when 3 of the 4 methods agreed on the local ancestry at that locus. We used the 1000 Genomes consensus ancestry calls in place of the true ancestry and r^2^ was calculated the same way as previously described. This is not a measure of accuracy since the true ancestry is not known, but a measure of calling consensus between our approach and other ancestry inference methods.

To check possible causes of poor correlation with the consensus calls, we selected a subset of 20 individuals from the real Mexican data, and determined CSVs using both the reference haplotypes as well as the regions of the remaining (not from the subset of 20) Mexican individuals' genotypes that are homozygous for a local ancestry. We then reran our method on the subset of Mexicans, first using both reference haplotypes and the held out Mexicans for training CSVs and second using only the reference haplotypes. We trained on the held out admixed genotypes by using the homozygous ancestry regions from 1000 Genomes local ancestry calls to identify new and spurious CSVs after training on the reference haplotypes. We see significant increases in accuracy when we do this, demonstrating poor reference panels as a major driver of the poor correlation.

### Ancestry calling for closely related populations

CSVs, as demonstrated in Table 1, contain sufficient information to distinguish continental groups from each other. However, it is possible to distinguish sub-continental populations from each other as well, such as distinguishing a JPT haplotype from a CHS haplotype, both of which are in the Asian continental group.

In order to distinguish sub-continental haplotypes we define sub-continental population-specific variants (sCSVs) as variants seen in one of the 1000 Genomes populations (e.g. GBR) but not in any of the other populations of all continental groups including its own. We perform a leave one out analysis where we remove one of the haplotypes from one of the populations, then train sCSVs on all remaining haplotypes. We then determine how many sCSVs from each of the populations we see on the held out haplotype.

We repeated this analysis, but instead of using the full haplotype to ask how many sCSVs are seen on each haplotype, we randomly choose sections from each haplotype between 0.05 and 30 megabases long and call the ancestral population of each haplotype segment as the population of which the most sCSVs were seen on the segment. With ten populations, random guessing results in an accuracy of 10%. We also calculate the accuracy of correctly calling the continental group from which each haplotype segment was drawn against the haplotype length.

In order to address the accuracy of sub-continental population calling in real data, we look at ASW individuals. In regions where the continental group ancestry (using the 1000 Genomes consensus calls) was called as African-African or European-European, we counted the number of sCSVs seen in each population. We normalized the counts to the number of observed sCSVs per megabase per haplotype. We compared these counts for the African-African ancestry regions to the expected number of observed sCSVs per megabase for a YRI haplotype and a LWK haplotype (calculated from the expected counts from the haplotypes used for Figure 6 which were then normalized by the length of the chromosome in mega-bases).

## Supporting Information

Figure S1 **Resolution in determining ancestry switch locations in LAMP-LD and Lanc-CSV.** For each true ancestry switch location in the simulated Puerto Rican data we calculated the distance in base pairs to the nearest inferred ancestry switch point for both LAMP-LD and Lanc-CSV from the true ancestry switch point. We only considered true switches where the inferred switches from both LAMP-LD and Lanc-CSV were less than 500 kb from the true switch point. The mean distance to the switch point for LAMP-LD was 91,145 bp and 75,644 bp for Lanc-CSV. For each true switch, we take the difference between the LAMP-LD error distance and Lanc-CSV's error distance and plot a histogram of these values. Positive values imply that at a true switch location LAMP-LD had greater error, negative values that our method had greater error; a zero value indicates that both methods are equally accurate.
(TIFF)

Figure S2
**The hidden Markov model for a 2-way admixed individual (e.g. African American).** The three types of states represent the three types of possible ancestry combinations: homozygous for African ancestry, homozygous for European ancestry or heterozygous for African and European ancestry. The probability of transitioning between the previous state and 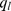 is a function of the genetic distance between the previous CSV and CSV_j_.(TIFF)

Figure S3 **Runtime (in CPU days) as a function of the number of individuals in a study with genotyping array data (and sequencing data for Lanc-CSV).** Lanc-CSV is always faster than LAMP-LD and MULTIMIX when run on either full genome sequencing data (see Figure 3 and Table S1) or genotyping array data. The full sequencing data contained ∼30 times more alleles than the genotyping array data. Only RFMix has comparable speed for full sequenced data and is faster for genotype array data. We show the runtime for RFMix with phasing time included.
(TIFF)

Software S1 **MATLAB code for running Lanc-csv.** Contained in the software package is the MATLAB code for running Lanc-csv on genotype data as well as a sample data set. The included README instructs the user on required input data and formatting. A C++ version of the code will additionally be available on our website: http://bogdan.bioinformatics.ucla.edu/software/lanccsv.
(ZIP)

Table S1 **Runtime in CPU days for LAMP-LD, MULTIMIX, RFMix and Lanc-CSV.** Runtimes were estimated by running each method on chromosome 10 in 200 individuals and extrapolated to full genome. Results are in total CPU days. All methods can be parallelized for proportional decreases in computing time. RFMix requires phased haplotype data and phasing time is reported in the parentheses.
(PDF)

Table S2 **Correlation of ancestry calls between our approach and the 1000 Genomes calls in real admixed individuals from 1000 Genomes.** Accuracy reported as r^2^ (haploid accuracy, diploid accuracy). The 1000 Genomes consensus local ancestry calls were made using LAMP-LD as one of the four methods. This demonstrates that poor accuracy is likely a result of poor reference panels.
(PDF)

Table S3 **Wahlund Effect on genotype probabilities.** When an allele has different frequencies in different populations and the populations are looked at as a single population, the Wahlund Effect predicts a decrease in heterozygosity. The magnitude of the effect decreases with the difference in the allele frequencies and with mixing between the populations. 98% of CSVs have an allele frequency <5%. Here we report the genotype probabilities assuming the admixed populations have established Hardy-Weinberg Equilibrium, and assuming they are completely unmixed (the most extreme version of the Wahlund Effect). We report these values for 10%, 50% and 80% admixture proportion of the CSV containing population. This demonstrates that the Wahlund Effect will have negligible effect on our method's performance.
(PDF)

Table S4 **Accuracy of Inferrence on 100 simulated admixed individuals among pairs of countries in Europe.** We used admixture proportions of (0.5,0.5) and 6 generations of admixture. Accuracy is reported as haploid error (see main text). We observe a high proportion of heterozygous ancestry calls (over 90%), consistent with increased ambiguity in the calling using sCSVs for closely related populations.
(PDF)
