# Supplementary figures and images for: Enhanced Methods for Local Ancestry Assignment in Sequenced Admixed Individuals
Source: PLoS Comput Biol. 2014 Apr 17;10(4):e1003555. doi: 10.1371/journal.pcbi.1003555 (PMC3990492; doi:10.1371/journal.pcbi.1003555)

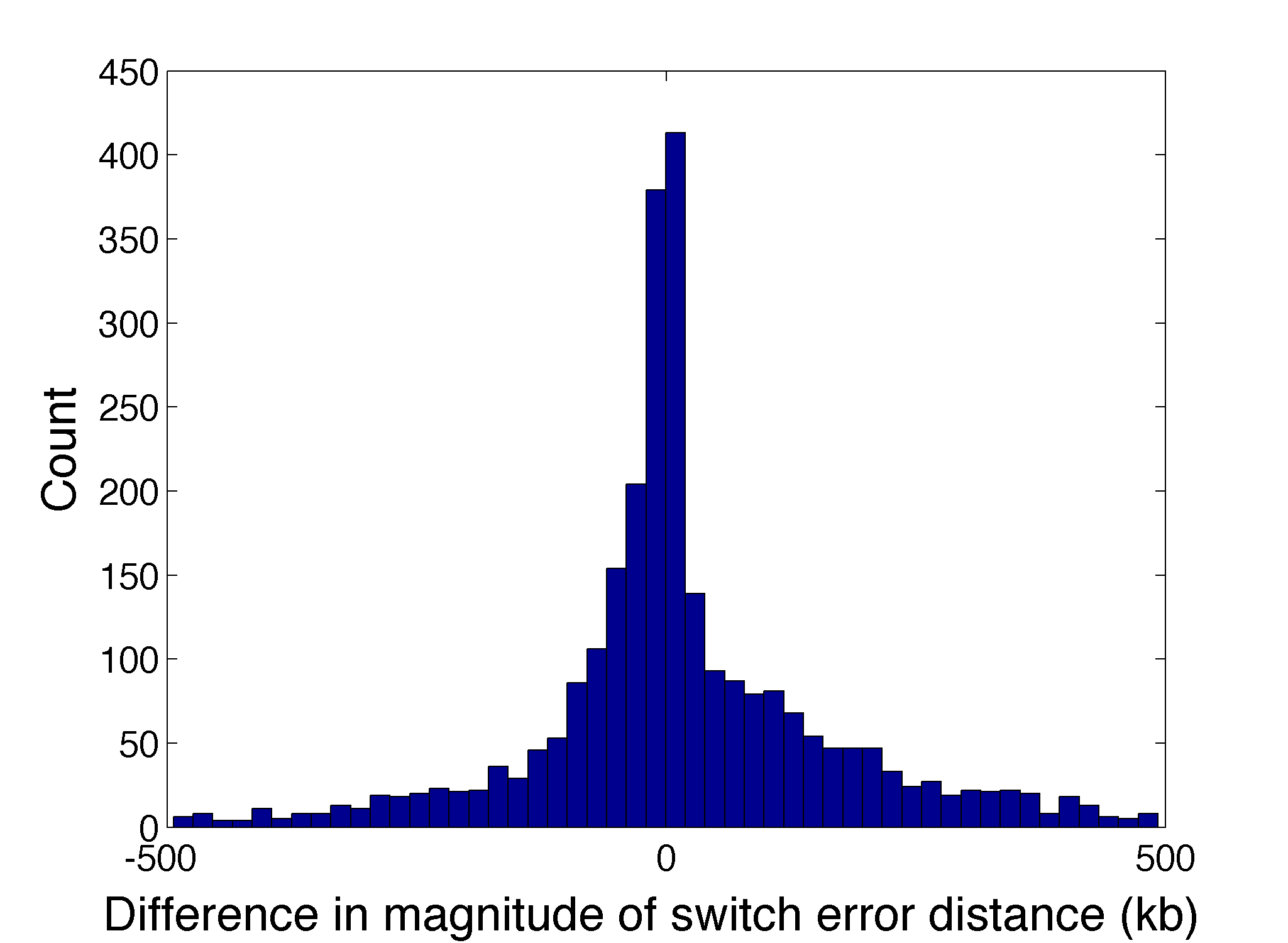

Supplement: Figure S1 — Resolution in determining ancestry switch locations in LAMP-LD and Lanc-CSV. For each true ancestry switch location in the simulated Puerto Rican data we calculated the distance in base pairs to the nearest inferred ancestry switch point for both LAMP-LD and Lanc-CSV from the true ancestry switch point. We only considered true switches where the inferred switches from both LAMP-LD and Lanc-CSV were less than 500 kb from the true switch point. The mean distance to the switch point for LAMP-LD was 91,145 bp and 75,644 bp for Lanc-CSV. For each true switch, we take the difference between the LAMP-LD error distance and Lanc-CSV's error distance and plot a histogram of these values. Positive values imply that at a true switch location LAMP-LD had greater error, negative values that our method had greater error; a zero value indicates that both methods are equally accurate. (TIFF) [file pcbi.1003555.s001.tif]

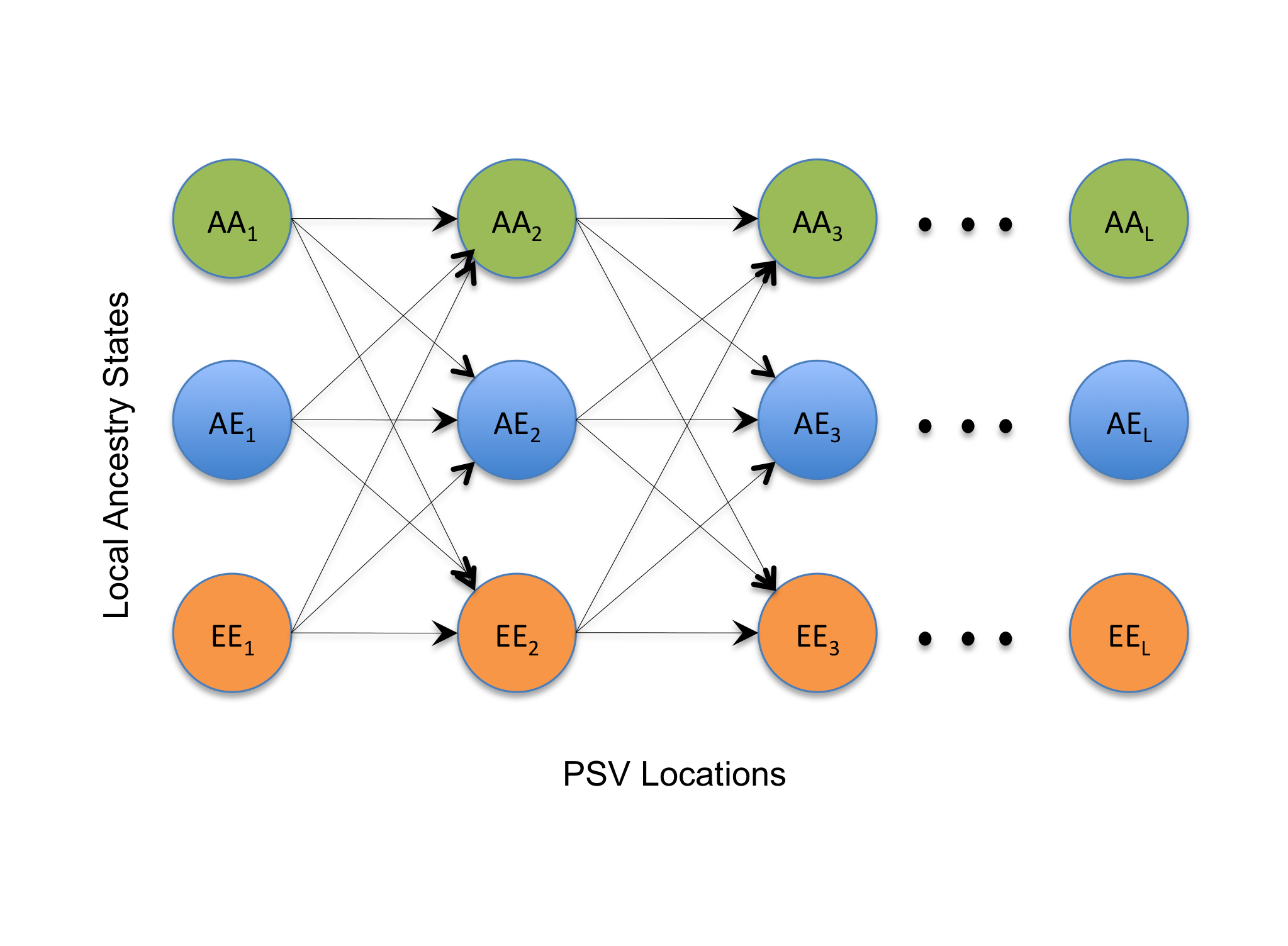

Supplement: Figure S2 — The hidden Markov model for a 2-way admixed individual (e.g. African American). The three types of states represent the three types of possible ancestry combinations: homozygous for African ancestry, homozygous for European ancestry or heterozygous for African and European ancestry. The probability of transitioning between the previous state and is a function of the genetic distance between the previous CSV and CSVj. (TIFF) [file pcbi.1003555.s002.tif]

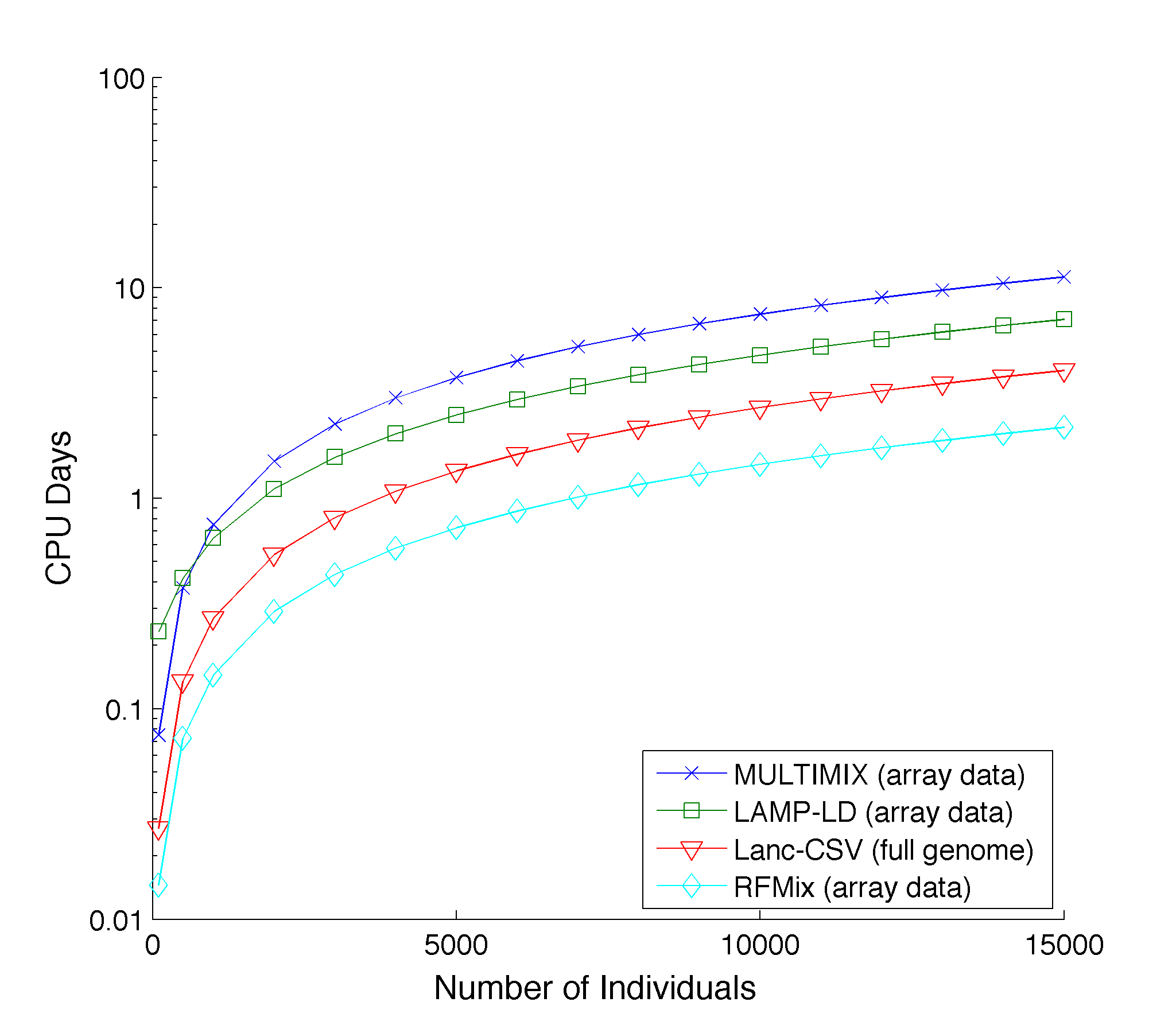

Supplement: Figure S3 — Runtime (in CPU days) as a function of the number of individuals in a study with genotyping array data (and sequencing data for Lanc-CSV). Lanc-CSV is always faster than LAMP-LD and MULTIMIX when run on either full genome sequencing data (see Figure 3 and Table S1) or genotyping array data. The full sequencing data contained ∼30 times more alleles than the genotyping array data. Only RFMix has comparable speed for full sequenced data and is faster for genotype array data. We show the runtime for RFMix with phasing time included. (TIFF) [file pcbi.1003555.s003.tif]
