# Supplementary material for: Enhanced Methods for Local Ancestry Assignment in Sequenced Admixed Individuals
Source: PLoS Comput Biol. 2014 Apr 17;10(4):e1003555. doi: 10.1371/journal.pcbi.1003555 (PMC3990492; doi:10.1371/journal.pcbi.1003555)
Supplement: Software S1 — MATLAB code for running Lanc-csv. Contained in the software package is the MATLAB code for running Lanc-csv on genotype data as well as a sample data set. The included README instructs the user on required input data and formatting. A C++ version of the code will additionally be available on our website: http://bogdan.bioinformatics.ucla.edu/software/lanccsv. (ZIP) [file pcbi.1003555.s004.zip › README.rtf]

In order to run this beta version of Lanc-CSV using the MATLAB code, the following are required for inference of 3-way admixture.  1) Population proportions:EuroProp=0.67WafrProp=0.20AsnProp=0.132) A vector of the allele frequencies in each of the three populations (e.g. Europeans, west Africans, and Asians). EuroMAFsWafrMAFsAsnMAFs3) a matrix containing the genotypes of the admixed individuals. The rows are the individuals and the columns are the genotypes.genotypes4) The number of generations since admixture.ngen=15 5) The number of Iterations to run of Lanc-CSV.numIterations=46) A vector containing the variant physical positions in base pairs.AllSNPPositionsARRAYExamples of all of this input data is contained in the PURSampleData.mat data structure.LancCSVrunner.m will run Lanc-CSV on the 5 example genotypes.  It will output a file called OutputData.mat which is an array, the same size as the genotypes array. Each variant position in each genome is assigned an ancestry in this output file.In this beta version the ancestry codes are as follows:1: European-European2: European-African3: European-Native American4: African-African5: African-Native American6: Native American-Native AmericanThe PURSampleData.mat structure also includes the true ancestry of the simulated genotypes at the variants in the SNPsOnChip array.The phased haplotypes for the simulated genotypes are also available in the PURSampleData.mat.The poisspdf.m function in the statistics toolbox is needed to run this script.
