# Supplementary material for: Enhanced Methods for Local Ancestry Assignment in Sequenced Admixed Individuals
Source: PLoS Comput Biol. 2014 Apr 17;10(4):e1003555. doi: 10.1371/journal.pcbi.1003555 (PMC3990492; doi:10.1371/journal.pcbi.1003555)
Supplement: Table S1 — Runtime in CPU days for LAMP-LD, MULTIMIX, RFMix and Lanc-CSV. Runtimes were estimated by running each method on chromosome 10 in 200 individuals and extrapolated to full genome. Results are in total CPU days. All methods can be parallelized for proportional decreases in computing time. RFMix requires phased haplotype data and phasing time is reported in the parentheses. (PDF) [file pcbi.1003555.s005.pdf]

Table S1: Runtime in CPU days for LAMP-LD, MULTIMIX, RFMix and Lanc-CSV.

	Runtime for 200 individuals on chromosome 10	Estimated runtime for 200 individuals on all chromosomes	Estimated runtime for 15000 individuals on all chromosomes
LAMP-LD (array data)	0.11	2.79	69.83
MULTIMIX (array data)	0.06	1.50	113.25
RFMix (array data)	0.004 (+0.01 for phasing)	0.11 (+0.18 for phasing)	8.06 (+13.123 for phasing)
LAMP-LD (full genome)	4.39	109.79	1,790.20
MULTIMIX (full genome)	3.30	82.58	6,193.80
RFMix (full genome)	0.03 (+0.22 for phasing)	0.79 (+5.5 for phasing)	59.46 (+412.5 for phasing)
Lanc-CSV	0.02	0.54	41.41

Runtimes were estimated by running each method on chromosome 10 in 200 individuals and extrapolated to full genome. Results are in total CPU days. All methods can be parallelized for proportional decreases in computing time. RFMix requires phased haplotype data and phasing time is reported in the parentheses.
