# Supplementary material for: Enhanced Methods for Local Ancestry Assignment in Sequenced Admixed Individuals
Source: PLoS Comput Biol. 2014 Apr 17;10(4):e1003555. doi: 10.1371/journal.pcbi.1003555 (PMC3990492; doi:10.1371/journal.pcbi.1003555)
Supplement: Table S2 — Correlation of ancestry calls between our approach and the 1000 Genomes calls in real admixed individuals from 1000 Genomes. Accuracy reported as r2 (haploid accuracy, diploid accuracy). The 1000 Genomes consensus local ancestry calls were made using LAMP-LD as one of the four methods. This demonstrates that poor accuracy is likely a result of poor reference panels. (PDF) [file pcbi.1003555.s006.pdf]

Table S2: Correlation of ancestry calls between our approach and the 1000 Genomes calls in real admixed individuals from 1000 Genomes.

	African American (ASW)	Mexican (MXL)	Puerto Rican (PUR)
LANC-CSV	0.94 (0.994 0.988)	0.63 (0.84, 0.68)	0.81 (0.96, 0.92)
LAMP-LD	99.12 (1, 1)	0.66 (0.89, 0.77)	0.79 (0.94, 0.79)

Accuracy reported as r^2 (haploid accuracy, diploid accuracy). The 1000 Genomes consensus local ancestry calls were made using LAMP-LD as one of the four methods. This demonstrates that poor accuracy is likely a result of poor reference panels.
