# Supplementary material for: Enhanced Methods for Local Ancestry Assignment in Sequenced Admixed Individuals
Source: PLoS Comput Biol. 2014 Apr 17;10(4):e1003555. doi: 10.1371/journal.pcbi.1003555 (PMC3990492; doi:10.1371/journal.pcbi.1003555)
Supplement: Table S3 — Wahlund Effect on genotype probabilities. When an allele has different frequencies in different populations and the populations are looked at as a single population, the Wahlund Effect predicts a decrease in heterozygosity. The magnitude of the effect decreases with the difference in the allele frequencies and with mixing between the populations. 98% of CSVs have an allele frequency <5%. Here we report the genotype probabilities assuming the admixed populations have established Hardy-Weinberg Equilibrium, and assuming they are completely unmixed (the most extreme version of the Wahlund Effect). We report these values for 10%, 50% and 80% admixture proportion of the CSV containing population. This demonstrates that the Wahlund Effect will have negligible effect on our method's performance. (PDF) [file pcbi.1003555.s007.pdf]

Table S3: Wahlund Effect on genotype probabilities

	Hardy-Weinberg Equilibrium with 10% admixture proportion	Unmixed 10% Admixture Proportion	Hardy-Weinberg Equilibrium with 50% admixture proportion	Unmixed 50% Admixture Proportion	Hardy-Weinberg Equilibrium with 80% admixture proportion	Unmixed 80% Admixture Proportion
P(G=0)	0.99	0.99	0.95	0.95	0.92	0.92
P(G=1)	0.01	0.01	0.05	0.05	0.08	0.08
P(G=2)	0.00	0.00	0.00	0.00	0.00	0.00

When an allele has different frequencies in different populations and the populations are looked at as a single population, the Wahlund Effect predicts a decrease in heterozygosity. The magnitude of the effect decreases with the difference in the allele frequencies and with mixing between the populations. 98% of CSVs have an allele frequency <5%. Here we report the genotype probabilities assuming the admixed populations have established Hardy-Weinberg Equilibrium, and assuming they are completely unmixed (the most extreme version of the Wahlund Effect). We report these values for 10%, 50% and 80% admixture proportion of the CSV containing population. This demonstrates that the Wahlund Effect will have negligible effect on our methods performance.
