# Supplementary material for: Enhanced Methods for Local Ancestry Assignment in Sequenced Admixed Individuals
Source: PLoS Comput Biol. 2014 Apr 17;10(4):e1003555. doi: 10.1371/journal.pcbi.1003555 (PMC3990492; doi:10.1371/journal.pcbi.1003555)
Supplement: Table S4 — Accuracy of Inferrence on 100 simulated admixed individuals among pairs of countries in Europe. We used admixture proportions of (0.5,0.5) and 6 generations of admixture. Accuracy is reported as haploid error (see main text). We observe a high proportion of heterozygous ancestry calls (over 90%), consistent with increased ambiguity in the calling using sCSVs for closely related populations. (PDF) [file pcbi.1003555.s008.pdf]

Table S4: Accuracy of Inferrence on 100 simulated admixed individuals among pairs of countries in Europe.

	FIN	GBR	TSI	CEU
FIN	na	0.76	0.77	0.77
GBR		na	0.74	0.75
TSI			na	0.77
CEU				na

We used admixture proportions of (0.5,0.5) and 6 generations of admixture. Accuracy is reported as haploid error (see main text). We observe a high proportion of heterozygous ancestry calls (over 90%), consistent with increased ambiguity in the calling using sCSVs for closely related populations.
